# Multi-Omics Analysis Reveals Changes in the Intestinal Microbiome, Transcriptome, and Methylome in a Rat Model of Chronic Non-bacterial Prostatitis: Indications for the Existence of the Gut-Prostate Axis

**DOI:** 10.3389/fphys.2021.753034

**Published:** 2022-01-11

**Authors:** Junsheng Liu, Yihe Wang, Guangwen Zhang, Liu Liu, Xichun Peng

**Affiliations:** ^1^Department of Food Science and Engineering, Jinan University, Guangzhou, China; ^2^Department of Biomedical Sciences, University of Copenhagen, Copenhagen, Denmark

**Keywords:** chronic non-bacterial prostatitis, DNA methylome, gut microbiota, gut-prostate axis, microbiome, transcriptome

## Abstract

Chronic non-bacterial prostatitis (CNP) is one of the most prevalent diseases in human males worldwide. In 2005, the prostate-gut axis was first proposed to indicate the close relationship between the prostate and the intestine. This study investigated CNP-induced changes of the gut microbiota, gene expression and DNA methylation in a rat model by using multi-omics analysis. Firstly, 16S rDNA sequencing presented an altered structure of the microbiota in cecum of CNP rats. Then, transcriptomic analysis revealed that the expression of 185 genes in intestinal epithelium was significantly changed by CNP. These changes can participate in the immune system, digestive system, metabolic process, etc. Finally, methylC-capture sequencing (MCC-Seq) found 73,232 differentially methylated sites (DMSs) in the DNA of intestinal epithelium between control and CNP rats. A combined analysis of methylomics and transcriptomics suggested an epigenetic mechanism for CNP-induced differential expression genes correlated with intestinal barrier function, immunity, metabolism, enteric infectious disease, etc. More importantly, the transcriptomic, methylomic and gut microbial changes were highly correlated with multiple processes including intestinal immunity, metabolism and epithelial barrier function. In this study, disrupted homeostasis in the gut microbiota, gene expression and DNA methylation were reported in CNP, which supports the existence of the gut-prostate axis.

## Introduction

Chronic non-bacterial prostatitis (CNP) is a common urinary disease, especially occurring in men below the age of 50. It is estimated that approximately 50% of all men suffer from prostatitis-like symptoms in their lives, and CNP accounts for 25% of all visits to urological clinics worldwide (Khan et al., 2017). Antibiotics, alpha-1 antagonists, anti-inflammatory agents, neuro-modulatory agents, and a multimodal approach are commonly employed as clinical therapies for CNP (Nickel, 2008). However, due to the heterogeneity of CNP, these management strategies are often ineffective, and this condition remains problematic to treat. Although infections, uric acid level, inflammation, autoimmunity or neuro-muscular mechanisms are all thought to be the etiologies, the pathogenesis of CNP is still controversial (Khan et al., 2017). Thus, providing a clear understanding of the pathogenic basis of CNP, as well as its complications, is critical for treatment and healthcare of this complex disease.

The intestine is the main site for digestion and absorption, and it is also the largest immune organ (Wittig and Zeitz, 2003). With the continuing progress of investigations, especially in the field of the gut microbiota, people are becoming increasingly aware of the health significance of the intestine (Schluter et al., 2020). Many studies have highlighted the concept of gut-organ axes and inter-organ communication between the intestine and other organs. For example, the gut microbiota is reported to regulate lung immunity and benefit respiratory health *via* the gut-lung axis (Wypych et al., 2019). Gut microbiota can play a role in the inflammation of alcoholic liver disease by activating toll-like receptor 4 through the gut-liver axis (Szabo, 2015). More importantly, *via* the interaction between nerve and immune cells, gut microbiota can also affect human mood, metabolism and behavior through the brain-gut-microbiota axis (Dinan and Cryan, 2017; Huh and Veiga-Fernandes, 2020). The concept of the gut-prostate axis was first proposed in 2005 to indicate the close relationship between the prostate and the intestine during the treatment of prostatitis (Yarnell and Abascal, 2005). Shoskes et al. (2016) reported that the gut microbiota of CNP patients showed lower alpha diversity and higher counts of *Varibaculum*. Konkol et al. (2019) found increase of *Rikenellaceae*, *Odoribacter*, and *Clostridiaceae*, as well as decrease of *Bacteroides uniformis*, *Lactobacillus*, and *Lachnospiraceae*, in the gut microbiota of CNP rats. In this study, 16S rDNA sequencing was performed to reveal the structural changes of the gut microbiota in CNP rats. Moreover, a combined analysis of transcriptomics and DNA methylomics was applied to explore CNP-induced changes in the intestine.

## Materials and Methods

### Animal Experiments

Twelve specific-pathogen-free (SPF) male Sprague Dawley (SD) rats (7–8 weeks, 180–200 g) were bought from Guangdong Medical Laboratory Animal Center, Guangdong, China). Rats were housed under controlled standard barrier conditions (temperature 23 ± 2°C, humidity 55% ± 5, and a 12 h light-dark cycle) at the Animal Center of Jinan University. After acclimatization for 1 week, rats were randomly divided into two groups (*n* = 6), and treated as follows: the ventral prostate of CNP rats (group CNP) was injected with 100 μL saline-dissolved 1% λ-carrageenan, and control rats (group CTL) were injected with equivalent saline (Wang W. et al., 2018). After 7 days, rats were anesthetized with pentobarbital sodium (50 mg/kg body weight) and sacrificed by exsanguination *via* the abdominal aorta.

All animal experiments were conducted with the approval of the Ethics Committee of Jinan University, China (No. IACUC-20190709-10). All aspects of the study were carried out in accordance with the European Community guidelines (Directive 2010/63/EU) for the care and use of experimental animals.

### Microbiome Sequencing and Data Analysis

16S rDNA sequencing and bioinformatics analysis were performed as previously described (Liu et al., 2020). In brief, the microbial genome DNA of the rat cecum contents was extracted and purified. The V4 region of 16S rDNA was amplified by PCR. After agarose gel electrophoresis, gel extraction, quantification, and library preparation, sequencing was performed on an Illumina HiSeq (read length: 250 bp and sequencing depth: 5w tags) with the MiSeq Reagent Kit. Bioinformatics analysis was performed using BMKCloud^1^ : the paired-end reads were assembled by FLASH v1.2.7 to produce raw tags. Then, raw tags with low quality ware removed by Trimmomatic v0.33 to get clean tags. UCHIME v4.2 was used to identify and filter the chimeric feature sequences. Next, 16S rDNA gene sequences were assigned to operational taxonomic units (OTUs) with pair-wise identity of more than 97% and taxonomically classified by Usearch. The generated OTUs were standardized based on the Greengenes data base, and the functions of OTUs were annotated and predicted based on Kyoto encyclopedia of genes and genomes (KEGG) and Cluster of orthologous groups (COG) data bases with PICRUSt v1.1.4. Partial least squares discrimination analysis (PLS-DA) with R v3.5.0 was applied to show the classification of different groups. The relative abundance of bacteria in each sample was obtained by analysis with QIIME2-2019.4, and figures were prepared with R v3.5.0. The LDA effect size (LEfSe) analysis was performed to identify the biomarkers for each group (significant when LDA score >3), and random-forest algorithm was applied with the random Forest v4.6.14 package.

### Transcriptome (mRNA) Sequencing and Data Analysis

Colonic epithelia (>95%) were obtained by scraping at 4°C (Kellermayer et al., 2011). Total RNA was extracted with the RNeasy mini kit (Qiagen, Germany). Paired-end libraries were synthesized using the TruSeq^®^ RNA Sample Preparation Kit (Illumina, United States) following the TruSeq^®^ RNA Sample Preparation Guide. In brief, the poly-A containing mRNA molecules were purified using poly-T oligo-attached magnetic beads. Following purification, the mRNA was fragmented into small pieces using divalent cations under 94°C for 8 min. The cleaved RNA fragments were copied into first strand cDNA using reverse transcriptase and random primers. This process was followed by second strand cDNA synthesis using DNA Polymerase I and RNase H. These cDNA fragments underwent an end repair process, the addition of a single “A” base, and ligation of the adapters. The products were purified and enriched with PCR to create the final cDNA library. Purified libraries were quantified by a Qubit^®^ 2.0 Fluorometer (Life Technologies, United States) and validated by an Agilent 2100 Bioanalyzer (Agilent Technologies, United States) to confirm the insert size and calculate the mole concentration. Cluster (*n* = 3) was generated by cBot with the library diluted to 10 pM and then was sequenced on the Illumina HiSeq 2500 (Illumina, United States; read length: 150 bp and sequencing depth: 6G) at Shanghai Biotechnology Corporation.

Raw reads were preprocessed with Seqtk to filter out sequencing adapters, short-fragment reads and other low-quality reads. Then, Hisat2 v2.0.4 was used to map the cleaned reads to the *Rnor 6.0.95* reference genome with two mismatches. After genome mapping, Cufflinks v2.1.1 was run with a reference annotation to generate FPKM values for known gene models. Differentially expressed genes were identified using Cuffdiff v2.2.1. The *p*-value significance threshold in multiple tests was set by the false discovery rate (FDR) (Benjamini and Yekutieli, 2001). The fold-changes were also estimated according to the FPKM in each sample. The differentially expressed genes were selected using the following filter criteria: *q*-value (adjusted *p*-value) ≦ 0.05 and fold-change ≧ 2. The differentially expressed genes were analyzed for gene set enrichment analysis (GSEA) with the KEGG databases.

### MethylC-Capture Sequencing and Data Analysis

Genome DNA of the colonic epithelia was extracted with gDNA Extraction Kit (Omega Bio-Tek Research, United States) and sheared to fragments of 150–200 bp (Covaris E-series Instrument, Australia). DNA libraries were prepared with SureSelect*^XT^* Methyl-seq Library Pre kit and then hybridized with hybridization reagents, blocking agents and the SureSelect capture library. Then, bisulfite conversion was performed by using EZ DNA Methylation-Gold Kit (Zymo Research, United States). The resulting DNA was desulphonated with a Zymo-Spin IC column and additional reagents from the EZ DNA Methylation-Gold Kit. After quality and quantity assessments (2100 Bioanalyzer, Agilent Technologies, United States), DNA samples were sequenced on the HiSeq X ten Platform (Illumina Technologies, United States; read length: 150 bp and sequencing depth: 10X) in the laboratory of the Shanghai Biotechnology Corporation (Shanghai, China).

The quality of raw data was assessed by FastQC v0.11.5 with the *Q* value: *Q* = −10 log_10_ (error ratio). The raw data were trimmed by Trim galore v0.4.1 to produce clean data (clean reads). In this step, the low-quality data (*Q* < 20, length < 70, etc.) and adaptors were excluded. The clean reads were processed by Bismark v0.15.0 for genome alignment (*Rat6*), and the PCR duplicates were removed. The information of cytosine (C), CpG C and methylated CpG C (5mC), as well as their distribution and frequency, can be extracted from the information of genome alignment. The reads with more than 10X read depth were processed by R methylKit v0.9.5 to indicate the details of the read depth and methylation level of all 5mC. The methylation level of 5mC between groups was compared and annotated by Bioconductor package, and the 5mC with mean methylation level difference >10% and nominal *p* < 0.05 between groups was considered to be differentially methylated site (DMS). The 5mC density (per million base) of each chromosome was calculated and statistically compared by independence-samples *t*-test (significant when *p* ≦ 0.05). According to the annotation of DMSs, all genes with DMSs in the promoter-TSS region were obtained for GSEA using the GO and KEGG databases online^2^. The possible changes in gene transcription can be predicted based on the methylation changes of promoter DNA.

## Results

### Chronic Non-bacterial Prostatitis Changed the Bacterial Structure of the Gut Microbiota

To investigate the effects of CNP on the gut microbiota, the cecum content (*n* = 6) was collected and sequenced on the Illumina HiSeq platform, and the composition of the microbial community was analyzed. By 16S rDNA sequencing, 757 and 758 operational taxonomic units (OTUs) were generated for the control (CTL) and CNP groups, respectively, and 754 OTUs were shared by them. Genome alignment found these OTUs belonged to 168 genera, which were all shared by the CTL and CNP groups. The alpha diversity of the gut microbiota in CNP patients was previously reported to be reduced (Shoskes et al., 2016). In this study, the cecum contents were collected for 16S rDNA sequencing at 7 days after surgery. The gut microbiota might have begun to recover and the alpha diversity (Shannon, Simpson, ACE and Chao indexes) of the gut microbiota were not found to be significantly changed (data were not shown).

**FIGURE 1:**
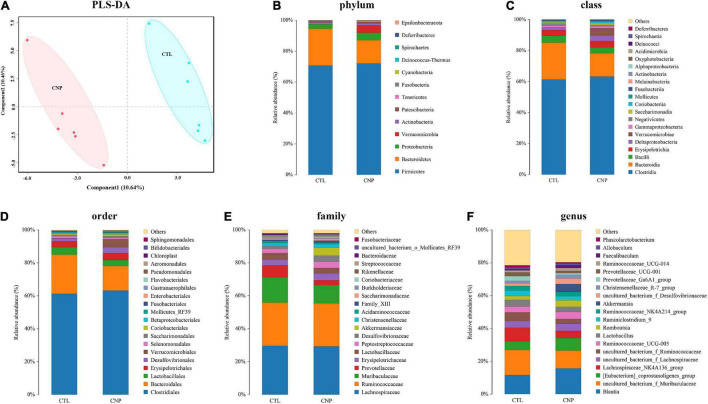
CNP-induced changes of gut microbiota. Rat cecum content was collected and sequenced, and the community structure was analyzed. PLS-DA analysis **(A)** was performed to show the changes in gut microbiota diversity between the CTL an CNP groups. The relative abundance of bacteria was compared at the phylum **(B)**, class **(C)**, order **(D)**, family **(E)**, and genus **(F)** levels.

**FIGURE 2:**
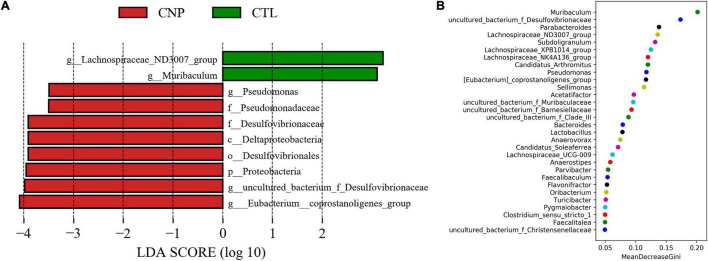
Bacterial markers in gut microbiota for CNP rats. LEfSe analysis **(A)** was performed to identify the markers (significant if LDA score >3) for CTL and CNP rats at different levels (phylum to genus). Furthermore, random-forest analysis **(B)** was conducted to investigated the difference at the genus level.

**FIGURE 3:**
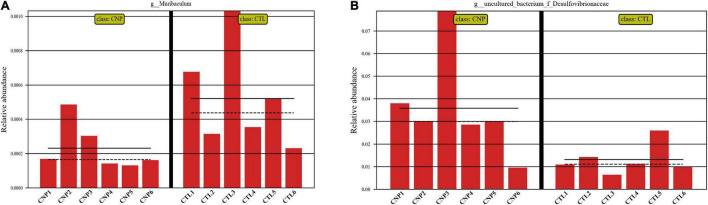
Relative abundance of *Muribaculum* **(A)** and *uncultured bacterium f Desulfovibrionaceae* **(B)** in CTL and CNP rats. The mean and median values of the relative abundance of each group were indicated with solid and dash lines, respectively.

Although CNP seemed to minimally influence the microbial OTUs and genera, partial least squares discrimination analysis (PLS-DA) presented that CNP rats were differently grouped from the CTL rats (Figure 1A), indicating a changed profile of the gut microbiota in CNP rats (statistical testing not performed). Histograms of relative abundance showed that the proportion of different bacteria was changed in the CNP group at different taxonomic levels (phylum to genus, Figures 1B–F). To identify the significantly changed taxa, linear discriminant analysis (LDA) effect size (LEfSe) analysis was performed (Figure 2A). The changes of different taxa were regarded as significant when LDA score was greater than 3. As Figure 2A shows, 8 taxa (1 phylum, 1 class, 1 order, 2 families, and 3 genera) were identified as bacterial markers for the CNP group, and 2 genera for the CTL group. The following analysis with random-forest algorithm method identified *Muribaculum* and *uncultured bacterium f Desulfovibrionaceae* as the genera with the highest importance score in the classifier (Figure 2B), which was consistent with the result of LEfSe analysis (Figure 2A). By comparing the relative abundance, we found a decrease of *Muribaculum* (Figure 3A) and an increase of *uncultured bacterium f Desulfovibrionaceae* (Figure 3A) in CNP rats. These bacteria might serve as diagnostic markers for CNP. These results demonstrated that CNP induced significant structural changes of the gut microbiota.

Different bacteria may interact with host in diverse ways and exert distinct functions. The functional changes of the gut microbiota in CNP rats were predicted with the KEGG and COG databases. Results showed that CNP might induce functional changes (not statistically different) in cell motility, transport and catabolism, infectious disease, extracellular structures, etc (Table 1). In this study, 16S rDNA sequencing could only allow structural analysis of the gut microbiota at genus level; metagenomics sequencing is required to investigate more detailed structural changes at species level and confirm the functional alterations.

**TABLE 1:** CNP was predicted to change functional pathways of gut microbiota.

Pathways	Relative abundance (%)		Changes (%)
	CTL	CNP	
KEGG: Cell motility	8.41E-1	7.71E-1	−8.31
KEGG: Transport and catabolism	2.91E-1	2.58E-1	−11.4
KEGG: Infectious diseases: Parasitic	6.79E-2	7.73E-2	13.9
KEGG: Digestive system	1.64E-2	1.25E-2	−24.0
KEGG: Excretory system	1.19E-2	1.52E-2	27.4
KEGG: Substance dependence	1.60E-3	8.74E-3	447
COG: Chromatin structure and dynamics	1.92E-2	2.81E-2	46.4
COG: Extracellular structures	7.40E-5	4.03E-5	−45.5
COG: RNA processing and modification	5.63E-3	7.51E-3	33.5

### Chronic Non-bacterial Prostatitis Changed the Transcriptome Profile of the Intestinal Epithelium

**FIGURE 4:**
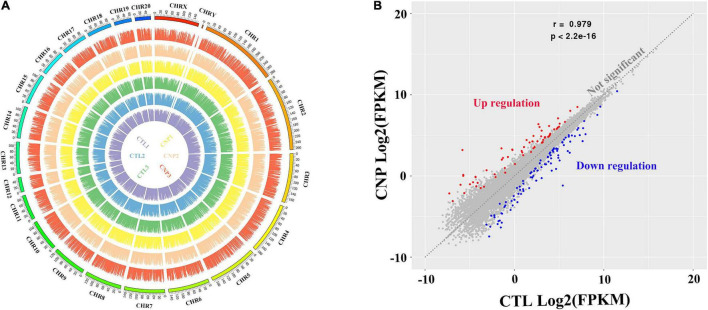
Gene expression profile **(A)** and differentially expressed genes in CNP rat epithelium **(B)**. Colonic epithelia of CTL and CNP rats were collected at sacrifice, and the gene expression profile was generated by transcriptome (mRNA) sequencing. CHR1-20, CHRX and CHRY stood for different chromosomes; the names of samples at the center had the same color with their patterns of expression; the height of each bar stood for the expression level **(A)**. The gene expression between group CTL and CNP was compared, and differentially expressed genes **(B)** were defined as *q* ≦ 0.05 and fold-change ≧ 2.

The gut microbiota can manipulate the expression of thousands of genes in colonic epithelial cells, and thus control intestinal homeostasis and inflammation (Ansari et al., 2020). To explore the CNP-induced changes of gene expression, colonic epithelial cells were collected, and the total mRNA was extracted. After reverse transcription, the cDNA was sequenced. Generally, by transcriptome sequencing averages of 44,617,848 and 43,329,914 clean reads were generated for each sample in the CTL and CNP groups, respectively. After genome mapping and statistical analysis, the relative abundance of mRNA and the position on the chromosomes of each sample are presented in Figure 4A. By comparing the relative abundance of mRNA, the expression of 185 genes was found to be significantly changed (*q* ≦ 0.05 and fold-change ≧ 2) in the CNP group (Figure 4B). Among these genes, 107 were downregulated and 78 were upregulated.

### Gene Set Enrichment Analysis Predicted Intestinal Dysfunctions in Chronic Non-bacterial Prostatitis Rats

**FIGURE 5:**
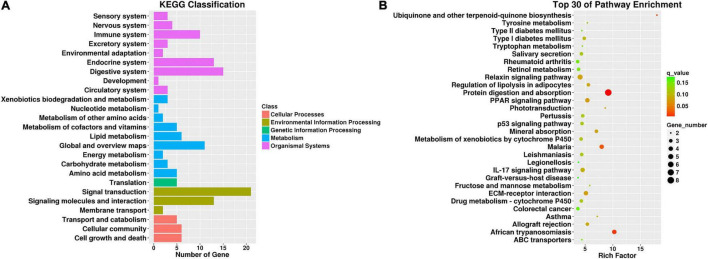
KEGG classification of differentially expressed genes between CNP and control rats **(A)** and top 30 enriched pathways **(B)**. All differentially expressed genes were classified by gene set enrichment analysis with the KEGG database **(A)**, and the top 30 enriched pathways are presented **(B)**.

To further interpret the physiological effects of the transcriptome changes, gene set enrichment analysis (GSEA) was performed based on the 185 changed genes in CNP rats using the KEGG database. KEGG classification (Figure 5A) showed that these genes participated in cellular processes, environmental information processing, genetic information processing, metabolism, and organismal systems. As presented in Figure 5A, many genes were involved in signal transduction, signaling molecules and interaction, digestive system, endocrine system, and immune system. These results indicated that CNP could affect intestinal functions through multiple approaches, including the nerve, endocrine and immune systems. In addition, CNP also influenced several metabolic processes, including carbohydrate, lipid, amino acid, and nucleotide metabolism (Figure 5A). The top 30 enriched KEGG pathways (Figure 5B) also showed that CNP might have an impact on intestinal processes of metabolism (protein digestion and absorption, mineral absorption, and fructose and mannose metabolism), immunity (PPAR signaling pathway, p53 signaling pathway, and IL-17 signaling pathway), and enteric disease (colorectal cancer, malaria, leishmaniasis, and legionellosis). All of these results suggested that CNP could cause extensive functional changes in the intestine and the occurrence of CNP might be accompanied by intestinal diseases.

### Chronic Non-bacterial Prostatitis Caused Abnormal Methylation of Intestinal DNA

**FIGURE 6:**
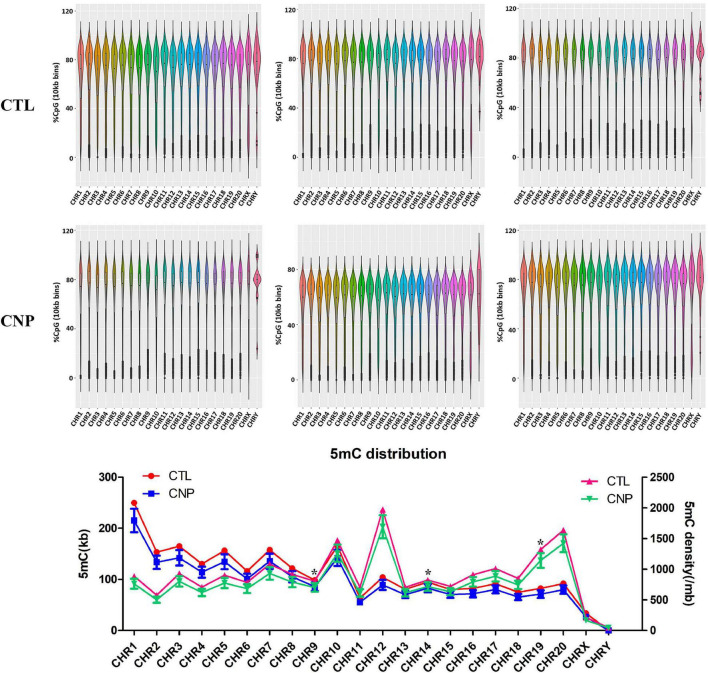
Distribution of 5mC between CNP and control rats. The methylation level of DNA was detected by methyl-capture sequencing and shown in the violin plot. The number of 5mC and the density (per million base) of each chromosome were calculated (*p ≦ 0.05).

DNA methylation is an epigenetic event in which a methyl group is covalently added to the DNA bases, typically the cytosine of CpG dinucleotides. DNA methylation can regulate gene expression without causing changes in the DNA sequence and is thought to play critical roles in the pathogenesis, progression and treatment of human diseases (Greenberg and Bourc’his, 2019). In this study, we investigated the effects of CNP on DNA methylation of intestinal epithelium by MethylC-Capture Sequencing (MCC-Seq). Generally, averages of 2,394,497 and 2,072,052 5-methylated cytosine (5mC) were generated for CTL and CNP groups, respectively. The number of 5mC in each chromosome and the density are shown in Figure 6. As Figure 6 displayed, the distribution of 5mC in each chromosome of the CNP group was less than that of the CTL group. This result indicated a demethylation effect of CNP. Notably, the cytosines in sex chromosomes (CHRX and CHRY) were less methylated, and CHR12 had the highest 5mC density (Figure 6). This result suggested that DNA methylation did not occur equally among chromosomes but with preference.

**FIGURE 7:**
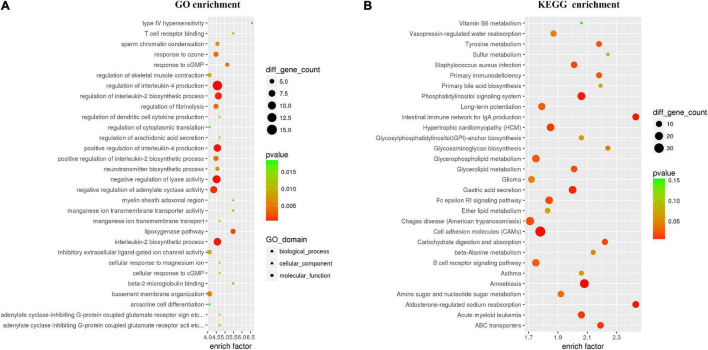
Top 30 processes generated by GO **(A)** and KEGG **(B)** enrichments based on genes with DMSs in the promoter-TSS region. All genes with DMSs in the promoter-TSS region were analyzed by gene set enrichment analysis with the GO and KEGG databases, and the top 30 enriched processes are presented.

**FIGURE 8:**
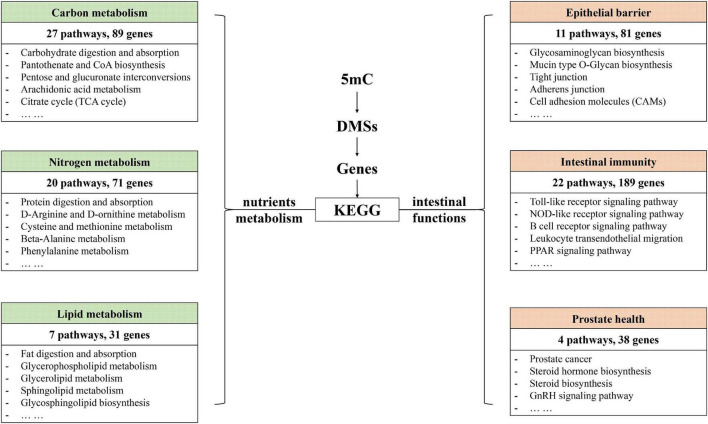
KEGG enrichment revealed possible functional changes occurring in CNP rat intestine due to DNA methylation changes.

To further explore the effects of CNP on DNA methylation, we compared the methylation level of all 5mC and found 73,232 differentially methylated sites (DMSs, mean methylation level difference >10% and *p* < 0.05). Among these DMSs, 60% were hypomethylated, which provided further evidence for the demethylation effect of CNP. DNA methylation may occur in different components of genes, and methylation in the promoter-transcription start site (promoter-TSS) region can inhibit the binding of transcription factors and subsequently regulate gene transcription, whereas the consequence of methylation in other regions remains an open question (Medvedeva et al., 2014). Annotation of the DMSs found 4,319 in the promoter-TSS region of 3,111 genes. GSEA with these genes generated 3,958 items (GO enrichment) and 212 pathways (KEGG enrichment). The top 30 processes (items or pathways) are shown in Figure 7. According to the result of KEGG enrichment, the methylation changes in the promoter-TSS region might participate in the process of carbon metabolism (27 pathways with 89 genes), nitrogen metabolism (20 pathways with 71 genes), and lipid metabolism (7 pathways with 31 genes, Figure 8). Moreover, KEGG enrichment also suggested several functional changes associated with epithelial barrier (11 pathways with 81 genes), intestinal immunity (22 pathways with 189 genes), and prostate health (4 pathways with 38 genes, Figure 8). All these results demonstrated that CNP might cause extensive abnormality of DNA methylation in the intestinal epithelium, and this might be an important way by which CNP induces abnormal gene expression *via* epigenetic mechanisms.

### Combination of Methylomic and Transcriptomic Analyses Suggested Epigenetic Mechanism for Chronic Non-bacterial Prostatitis-Induced Dysfunction in Gene Expression

**FIGURE 9:**
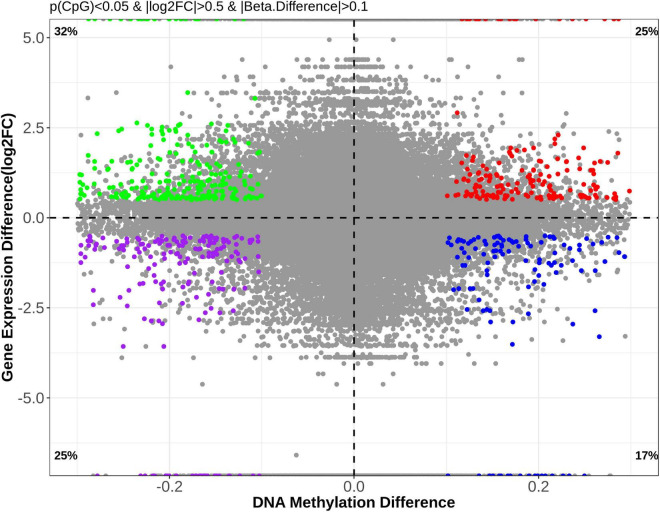
Correlational analysis of DNA methylation and gene expression. To investigate the possible effects of DNA methylation changes on gene expression, genes with | methylation difference| > 0.1 (*p* < 0.05) and | log2FC| > 0.5 (*p* < 0.05) were selected for further analysis and are presented.

DNA methylation is an important regulator for gene expression. A combined analysis of the DNA methylome and transcriptome (Figure 9) was performed to probe into the epigenetic regulations on gene expression. The genes and DMSs (in the promoter-TSS region) were selected when | gene expression difference (log2FC)| > 0.5 with *p* < 0.05 and | DNA methylation difference| > 0.1 with *p* < 0.05, which are highlighted in Figure 9. Because hypermethylation in the promoter-TSS is linked to gene silencing while hypomethylation causes gene overexpression, we finally obtained 316 genes that were supposed to be regulated by DNA methylation. Among them, 202 genes were upregulated (green dots in Figure 9), and 114 genes were downregulated (blue dots in Figure 9). The much greater amount of upregulated genes was consistent with the demethylation effect of CNP (Figure 6). In this study, *p* < 0.05 was used for the judgment of DMSs to find out the putative modified genes by DNA methylation. To study the possible effects of DNA methylation on the gut, KEGG enrichment was performed with these 316 genes. By screening all of the 99 pathways generated by KEGG enrichment, we found that DNA methylation might manipulate intestinal barrier function by modifying the cell adhesion molecules (five genes upregulated and three genes downregulated) and tight junction (three genes upregulated, Table 2) in CNP rats. We also presented the pathways associated with intestinal immunity, metabolism, and infectious disease, and detailed the expression changes of the involved genes (Table 2). The alteration in intestinal barrier function might cause deviant host interaction with the luminal environment, including intestinal microbes and their metabolites, which could further influence intestinal immunity, metabolism, and enteric infectious disease. Notably, the result of KEGG enrichments of the DNA methylome (Figure 8) showed much similarity with its combined analysis with the transcriptome (Table 2) in metabolism, intestinal immunity, epithelial barrier function, etc. These results suggested the great possibility of DNA methylation in participating CNP-induced gene expression changes.

**TABLE 2:** Abnormal DNA methylation-associated intestinal functions and gene expression changes.

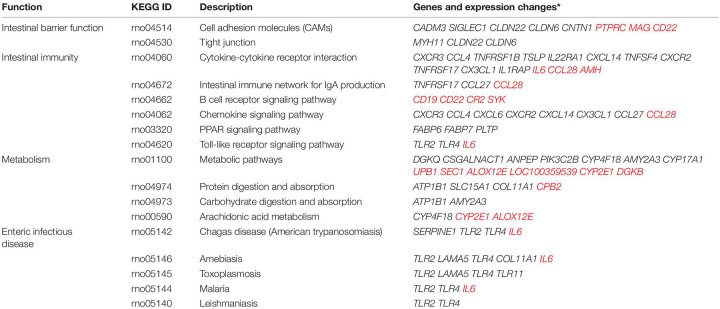

*Gene names in black: expression upregulated; gene names in red: expression downregulated.

## Discussion

Prostatitis is the most common urological problem in men under the age of 50, and the third most common problem in men over the age of 50 (Smieško, 2020). Antibiotics are commonly used for clinical treatment of prostatitis. Studies document that patients treated with long-term powerful antibiotics develop dysbiosis or abnormal gut flora (Bergman et al., 1989). A pilot study suggests that intestinal bacterial overgrowth and irritable bowel syndrome are common in patients with chronic prostatitis (Weinstock et al., 2011). Shoskes et al. (2016) analyzed the gut microbiome of patients with prostatitis and found an overgrowth of *Varibaculum*. Moreover, an *in vivo* experiment indicates a close relationship between changes of the gut microbiota and improvement of CNP (Konkol et al., 2019).

In this study, the expression of 185 genes of the colonic epithelium was found to be significantly changed in CNP rats (Figure 4B) using mRNA sequencing. KEGG classification with these genes indicated intestinal problems in the digestive, metabolic, immune, and excretory processes (Figure 5A). Changes in nutrient digestibility and the metabolic network (Supplementary Figure 1) might build a fundamental basis for the changes in the gut in CNP rats. Digestive and metabolic changes in the intestine have a high possibility of affecting the nutrient availability in the luminal content, and thus modifying the abundance of specific bacteria (Figure 1). Previous study showed that high-fat diet caused enrichment of *uncultured bacterium f Desulfovibrionaceae* in the cecum and colon (Wang et al., 2020). Similarly, a high-fat sucrose Western-style diet was found to increase the abundance of this bacteria (Tachon et al., 2014). Our results presented a significant increase of *uncultured bacterium f Desulfovibrionaceae* in CNP rats (Figure 3B), which offered further evidence for the linkage between intestinal bacterial overgrowth and irritable bowel syndrome in CNP patients (Weinstock et al., 2011). Moreover, the abundance of *Desulfovibrionaceae* is reported to be positively correlated with the duration of poly-autoimmunity (Bibbo et al., 2020). All these results suggested that overgrowth of *uncultured bacterium f Desulfovibrionaceae* might an important indicator for disease conditions. It has been reported that the relative abundance of *Lactobacillus* was decreased in CNP rats (Konkol et al., 2019). In this study, the relative abundance of *Lactobacillus* was also found to be decreased from 4.0 to 3.2% in CNP rats (Figure 1F). The changes of the gut microbiota might further contribute to the alteration in immune status of the host intestine (Table 2). As predicted with different databases, CNP might alter the functions of gut microbiota in the digestive system, excretory system, etc (Table 1). This result indicated that CNP-induced dysbiosis might in turn exert an effect on the intestine (Figure 5) as well as other organs. The identified bacteria in Figures 2, 3 are of great potential to be used for the discrimination of CNP and conventional rats. More importantly, biomarkers in the gut microbiota of the CNP patients can be identified in the same way and potentially applied for the clinical diagnosis of CNP.

Notably, with the KEGG database, we found that CNP-shaped gut microbiota was linked to parasitic disease (Table 1) and CNP-induced changes in gene expression were also associated with malaria (Figure 5B). A case of malarial prostatitis was reported decades ago in 1925 (Chopra, 1925). In 2012, the association of *Leishmania spp*. infection and chronic prostatitis was reported for the first time in a dog (Mir et al., 2012). However, no data are available on whether parasitic infections affect CNP or if CNP patients are more susceptible to parasitic infections.

The intestinal mucus is an organized glycoprotein network with a specific glycan structure. Covering the surface of the epithelium, intestinal mucus forms a physical barrier to prevent translocation of invading pathogens as well as commensal bacteria (Martens et al., 2018). Beyond its defensive function, intestinal mucus can also feed certain intestinal bacteria as a nutrient source. Thus, the mucus layer contributes to bacterial colonization, and changes in the mucus composition can induce a difference in gut microbiota (Sommer et al., 2014). O-linked glycan is estimated to contribute approximately 80% to the total molecular weight of intestinal mucus (Marcobal et al., 2013). MCC-Seq showed that CNP induced epigenetic changes associated with glycosaminoglycan biosynthesis and mucin type O-Glycan biosynthesis (Figure 8). This observation indicated that CNP might change the composition of the intestinal mucus and thus contribute to the changes of specific intestinal bacteria (Figures 2, 3) and modify the interaction of gut microbiota (Supplementary Figure 2). As a mediator for the interaction between gut microbiota and the host, the intestinal mucus is of vital importance in maintaining normal intestinal epithelial barrier function. It is reported that dysbiosis of gut microbiota can lead to degradation of the colonic mucus barrier and thus promote pathogen susceptibility (Desai et al., 2016). By MCC-Seq, in combination with mRNA sequencing (Table 2), we found clues for dysfunctions of the cell adhesion molecules and tight junctions in CNP rats (Figure 8). These results indicated disruption in the intestinal barrier function due to glycan-induced bacterial changes, which might further contribute to the translation of luminal microbes and thus change the immune status of the intestine (Figure 8 and Table 2) and change the susceptibility to parasitic infections (Figure 7 and Table 2). Additionally, the modified profile of the gut microbiota might also contribute to the susceptibility of infection (Table 1).

KEGG enrichment indicated that DNA methylation changes in CNP rats might affect several processes associated with prostate health, including prostate cancer (rno05215), steroid biosynthesis (rno00100), steroid hormone biosynthesis (rno00140), GnRH signaling pathway (rno04912), etc (Figure 8). GSEA on the differentially expressed genes also revealed the influence of CNP on steroid hormone biosynthesis (KEGG: rno00140), response to corticosteroid (GO: 0031960), response to steroid hormone (GO: 0048545), steroid metabolic process (GO: 0008202), cellular response to corticosteroid stimulus (GO: 0071384) and cellular response to steroid hormone stimulus (GO: 0071383). Androgen and estrogens are both steroid hormones. The age-related prevalence of CNP is reported to be associated with a decrease in the testosterone to estradiol ratio (Bernoulli et al., 2008). The disorder in androgen production in turn exacerbates inflammation in the prostate (Meng et al., 2011). Our results indicated that the CNP-induced disorder in hormone production might be promoted by the abnormality of several intestinal processes associated with steroids.

In previous studies, aberrant CpG hypermethylation always contributed to the development of diseases, and demethylation exerts reversing effects (Cao et al., 2016; Li et al., 2018; Wang G. et al., 2018). In this study, DNA hypomethylation (Figure 6), accompanied by overexpression of 202 genes (Figure 9), was observed in colonic epithelial cells of CNP rats. In this study, the DMSs was defined as | DNA methylation difference| > 0.1 with *p* < 0.05. A more stringent threshold, such as *q* < 0.05 might generate less overlapping genes in transcriptome and methylome analysis. Here, *p* < 0.05 was used to find out more putative DNA methylation-regulated genes. It deserves further investment to determine whether the expression of these overlapping genes was regulated by DNA methylation, and whether the effects of the disease on DNA methylation vary between organs. First, the DMSs identified by methylC-capture sequencing should be confirmed by pyrosequencing. Second, the DEGs identified by transcriptome sequencing should be confirmed by q-PCR or d-PCR. Moreover, further analysis should be performed to investigate whether the gene transcription was influenced by DNA methylation. The aberrant DNA methylation provided a putative epigenetic mechanism for CNP-induced differentially expressed genes.

Here, we performed a pilot study of the effects of CNP on the gut with multi-omics analysis in a rat model. Our results showed that carrageenan injection into the prostate gland caused disorders in bacterial structure of the gut microbiota, gene expression, and DNA methylation. All these results support the existence of the gut-prostate axis and suggest that special attention should be focused on alleviating intestinal complications when treating CNP. Several limitations of the present study deserve further investigation. The differentially expressed genes in CNP rats should be confirmed by real-time PCR or digital PCR, and KEGG predicted functional dysfunctions should be investigated by real-time PCR and western blot to provide more convincing evidence for the existence of CNP; the changes of the gut microbiota at species level as well as their functional genes associated with carbohydrate-active enzymes, antibiotic resistance and virulent factors can be analyzed by a genome-wide sequencing; moreover, metabolomics analysis can link the changes in gut microbiome with transcriptome.

## Data Availability Statement

The datasets presented in this study can be found in online repositories. The names of the repository/repositories and accession number(s) can be found below: https://www.ncbi.nlm.nih.gov/, GSE179639 and https://www.ncbi.nlm.nih.gov/, GSE159440.

## Ethics Statement

The animal study was reviewed and approved by the Ethics Committee of Jinan University.

## Author Contributions

JL and YW designed the study, performed the research, analyzed the data, and prepared the manuscript. GZ and LL performed the research and interpreted the results. XP supervised the study and revised the manuscript. All authors contributed to the article and approved the submitted version.

## Conflict of Interest

The authors declare that the research was conducted in the absence of any commercial or financial relationships that could be construed as a potential conflict of interest.

## Publisher’s Note

All claims expressed in this article are solely those of the authors and do not necessarily represent those of their affiliated organizations, or those of the publisher, the editors and the reviewers. Any product that may be evaluated in this article, or claim that may be made by its manufacturer, is not guaranteed or endorsed by the publisher.

## Footnotes

1
www.biocloud.net

2
http://enrich.shbio.com/

## Supplementary Material

Supplementary Figure 1 CNP-induced gene expression changes in the network of metabolic process. Upregulated genes are shown in red and downregulated genes are shown in green.

Supplementary Figure 2 Interactions of gut microbiota at genus level. **(A)** Group CTL; **(B)** Group CNP.
