# Supplementary figures and images for: Multi-Omics Analysis Reveals Changes in the Intestinal Microbiome, Transcriptome, and Methylome in a Rat Model of Chronic Non-bacterial Prostatitis: Indications for the Existence of the Gut-Prostate Axis
Source: Front Physiol. 2022 Jan 11;12:753034. doi: 10.3389/fphys.2021.753034 (PMC8787367; doi:10.3389/fphys.2021.753034)

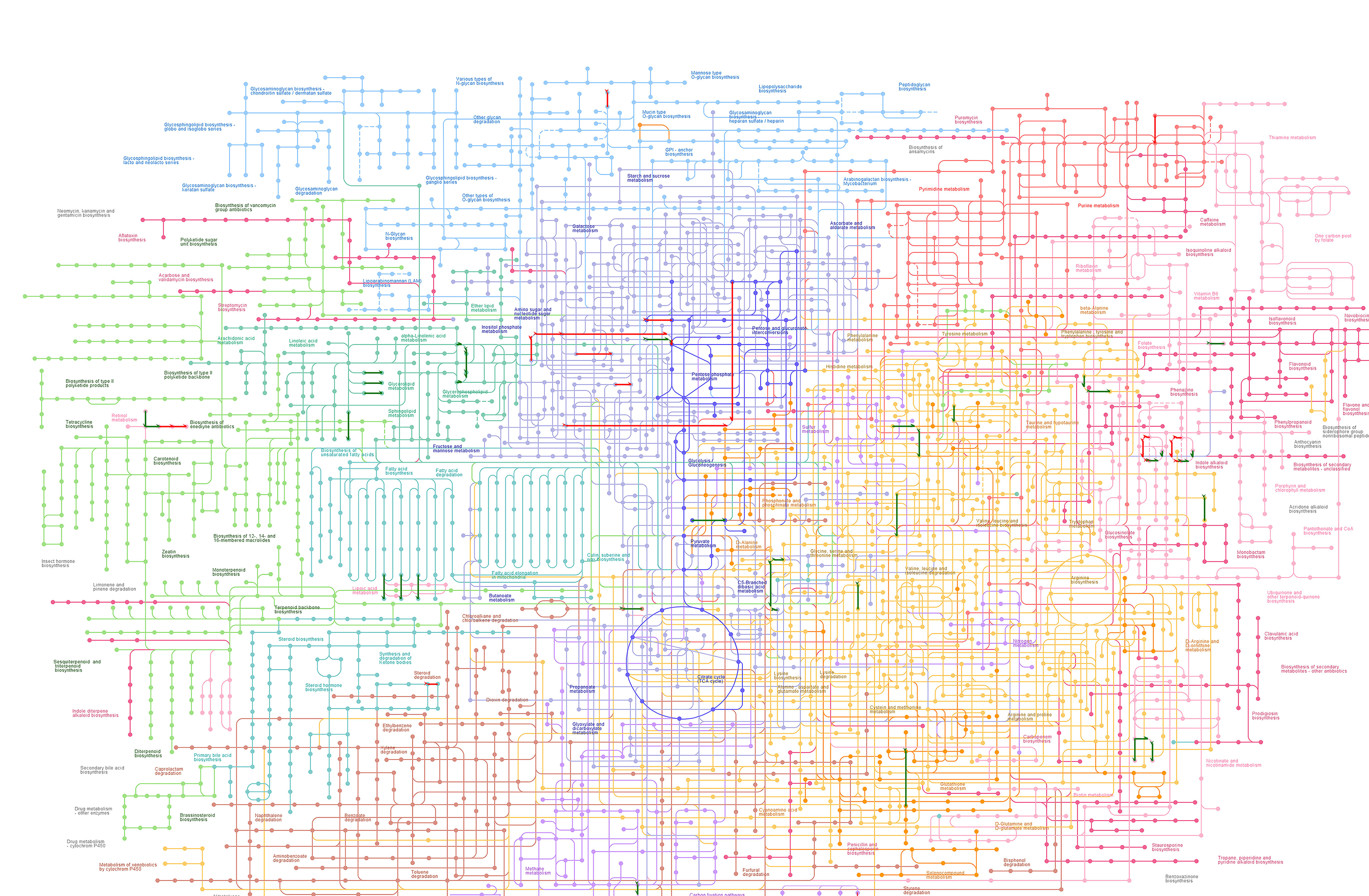

Supplement: Supplementary Figure 1 — CNP-induced gene expression changes in the network of metabolic process. Upregulated genes are shown in red and downregulated genes are shown in green. [file Image_1.JPEG]

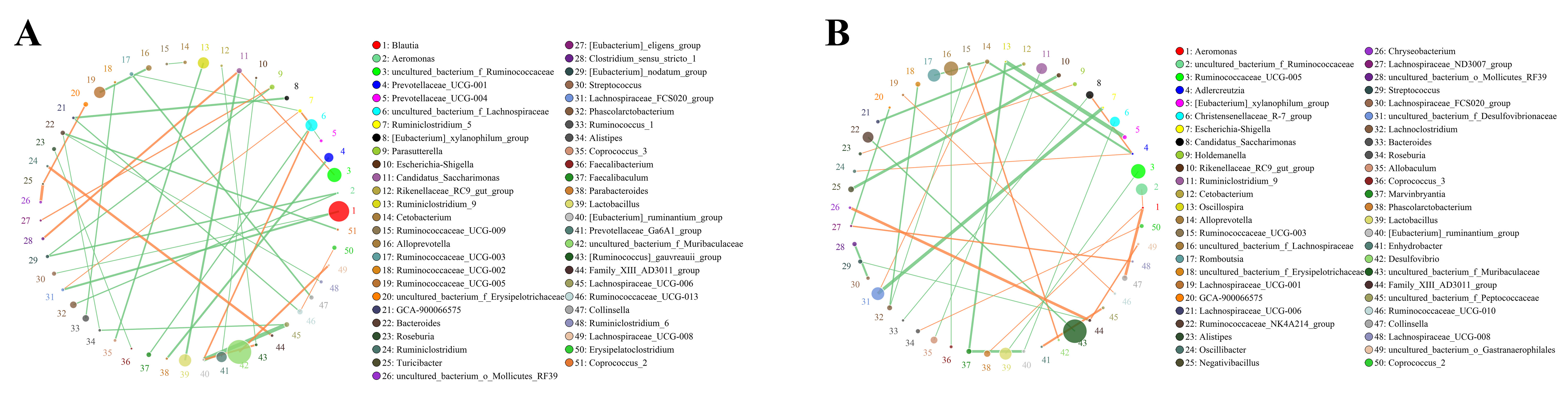

Supplement: Supplementary Figure 2 — Interactions of gut microbiota at genus level. (A) Group CTL; (B) Group CNP. [file Image_2.JPEG]
